# Evaluation of lung recovery after static administration of three different perfluorocarbons in pigs

**DOI:** 10.1186/2050-6511-15-53

**Published:** 2014-09-25

**Authors:** Mourad Chenoune, Ludovic De Rochefort, Patrick Bruneval, Fanny Lidouren, Matthias Kohlhauer, Aurélien Seemann, Bijan Ghaleh, Matthias Korn, Rose-Marie Dubuisson, Anis Ben Yahmed, Xavier Maître, Daniel Isabey, Jean-Damien Ricard, Richard E Kerber, Luc Darrasse, Alain Berdeaux, Renaud Tissier

**Affiliations:** 1 INSERM U955, Equipe 3, Créteil F-94010, France; 2 Université Paris-Est, UMR_S955, UPEC, Créteil F-94000, France; 3 Université Paris-Est, Ecole Nationale Vétérinaire d’Alfort, Maisons-Alfort F-94704, France; 4 IR4M (Imagerie par Résonance Magnétique Médicale et Multi-modalités), Univ Paris-Sud, CNRS, UMR8081, Orsay, France; 5 INSERM U970, Paris F75015, France; 6 INSERM U955, Equipe 13, Créteil F-94010, France; 7 INSERM, IAME, UMR 1137, F-75018 Paris, France; 8 Univ Paris Diderot, IAME, UMR 1137, Sorbonne Paris Cité, F-75018 Paris, France; 9 AP-HP, Service de Réanimation Médico-chirurgicale, Hôpital Louis Mourier, F-92700 Colombes, France; 10 Department of Internal Medicine, Division of Cardiovascular Medicine, University of Iowa Hospitals and Clinics, Iowa City IA 52242, US; 11 INSERM U955, Equipe 3, Ecole Nationale Vétérinaire d’Alfort, 7, avenue du Général de Gaulle, 94704 Maisons-Alfort cedex, France; 12 Current address: Cardiovascular Department, AP-HP - University Hospital Henri Mondor, F-94000 Creteil, France

**Keywords:** Perfluorocarbon, Liquid ventilation, Macrophage, Swine

## Abstract

**Background:**

The respiratory properties of perfluorocarbons (PFC) have been widely studied for liquid ventilation in humans and animals. Several PFC were tested but their tolerance may depend on the species. Here, the effects of a single administration of liquid PFC into pig lungs were assessed and compared. Three different PFC having distinct evaporative and spreading coefficient properties were evaluated (Perfluorooctyl bromide [PFOB], perfluorodecalin [PFD] and perfluoro-N-octane [PFOC]).

**Methods:**

Pigs were anesthetized and submitted to mechanical ventilation. They randomly received an intra-tracheal administration of 15 ml/kg of either PFOB, PFD or PFOC with 12 h of mechanical ventilation before awakening and weaning from ventilation. A Control group was submitted to mechanical ventilation with no PFC administration. All animals were followed during 4 days after the initial PFC administration to investigate gas exchanges and clinical recovery. They were ultimately euthanized for histological analyses and assessment of PFC residual concentrations within the lungs using dual nuclei fluorine and hydrogen Magnetic Resonance Imaging (MRI). Sixteen animals were included (4/group).

**Results:**

In the PFD group, animals tended to be hypoxemic after awakening. In PFOB and PFOC groups, blood gases were not significantly different from the Control group after awakening. The poor tolerance of PFD was likely related to a large amount of residual PFC, as observed using MRI in all lung samples (≈10% of lung volume). This percentage was lower in the PFOB group (≈1%) but remained significantly greater than in the Control group. In the PFOC group, the percentage of residual PFC was not significantly different from that of the Control group (≈0.1%). Histologically, the most striking feature was an alveolar infiltration with foam macrophages, especially in the groups treated by PFD or PFOB.

**Conclusions:**

Of the three tested perfluorocarbons, PFOC offered the best tolerance in terms of lung function, gas exchanges and residuum in the lung. PFOC was rapidly cleared from the lungs and virtually disappeared after 4 days whereas PFOB persisted at significant levels and led to foam macrophage infiltration. PFOC could be relevant for short term total liquid ventilation with a rapid weaning.

## Background

Liquid ventilation with perfluorocarbons (PFC) has been proposed to improve gas exchanges and lung compliance in adults [1,2] and infants [3] with acute respiratory distress. Chemically, these liquids are fluorinated organic compounds with high density, low surface tension and great solubility for oxygen and carbon dioxide [4] as compared to blood or water. During “total liquid ventilation”, lungs are filled with liquid PFC and ventilated with liquid tidal volumes [5]. This requires specific ventilators not yet tested in humans. An easier approach has been formerly tested using “partial liquid ventilation” in which lungs were partially filled with PFC (approximately up to the functional residual capacity) and conventionally ventilated with gas ventilation [5].

In animals, different PFC have been tested for partial and/or total liquid ventilation, including Rimar 101 [6-8], FC-77 [9], perfluorodecalin (PFD) [3,10] or perfluorooctyl bromide (PFOB), which was the most widely investigated [2,10-13]. In humans, PFOB was investigated for its excellent spreading properties and low vapor pressure allowing homogenous pulmonary repartition and low replacement rate for prolonged episodes of partial liquid ventilation. Despite promising preliminary results [2,12], this was not shown to improve outcome after acute respiratory distress syndrome in adults [14].

Another possible application for total liquid ventilation with PFC is ultra-fast induction of hypothermia [8,15-18]. In this application, PFC are used as cooling liquids and the lung as a thermal exchanger. This technique requires dedicated liquid ventilators [8,19-21] and can induce ultra-fast cooling and potent cardiac and neurological protection after cardiac arrest in animals [8,9,15-17,21,22]. In this indication, total liquid ventilation is expected to be very short and used only for induction of hypothermia (<30 min). The protective effects of hypothermic liquid ventilation have been extensively shown in rabbits [8,15,22] but need now to be confirmed in pigs. These species are indeed considered as a gold-standard for all confirmation studies in the field of cardiac arrest. However, pigs were shown to be very sensitive to some PFC as macrophages infiltration could compromise lung recovery after liquid ventilation with FC-77 [9]. Our present goal was to investigate the tolerance in pigs of several other PFC approved for a medical use in humans.

Accordingly, the effect of several PFC, including liquids with distinct evaporative and spreading coefficient properties (PFOB, PFD and perfluoro-N-octane [PFOC]), were properly assessed in pigs. To evaluate the intrinsic effect of these PFC, they were infused in a single and static administration within the lungs, with no further total liquid ventilation or hypothermia. We choose this design for two reasons: 1) it resembles to the weaning phase that follows liquid ventilation, without any other confounding factor related to the putative effects induced by liquid ventilation; 2) data suggests that this transition phase where liquid coexist with gas ventilation, exposes the most lungs to possible injury. Lung recovery was assessed by gas exchanges and histological appearance after 4 days of follow-up. Importantly, residual PFC distribution within the lungs was also assessed using an original clinically-relevant tool with fluorine magnetic resonance imaging (MRI) technique [23-25].

## Methods

This study was conducted in accordance with French regulations after approval by the local ethical committee (Ethical committee #16 of the “*Comité National de Réflexion Ethique sur l’Expérimentation Animale*”; Additional file 1). All experiments were performed in laboratory female pigs crossed between Large White and Landrace (≈25 kg and ≈ 3 months), after an appropriate control of the clinical status.

### Animal preparation

Pigs were anesthetized using ketamine (20 mg/kg i.m.), acepromazine (0.25 mg/kg i.m.) and pentobarbital (10 mg/kg/h i.v.). They were intubated and mechanically ventilated with a volume-controlled ventilator (Alpha Vet, Minerve, France; FiO_2_ = 30%). Respiratory rate and tidal volume were set at 17 cycles/min and ≈ 330-350 ml/cycle, respectively. Left carotid artery and left external jugular vein catheters were inserted for blood pressure measurement and sampling and drug administration, respectively. End-tidal CO_2_ concentration in the expired air (EtCO2) and blood oxygen saturation (SpO2) were continuously monitored (Mindray PM-8000 Vet, Hamburg, Germany). We also recorded electrocardiogram as well as rectal and esophageal temperatures and measured arterial blood pH, O_2_ and CO_2_ partial pressures (PaO_2_ and PaCO_2_, respectively). After surgery, animals received antibiotics (amoxicillin; 15 mg/kg s.c.) and analgesia (meloxicam; 0.4 mg/kg/day i.m.). Throughout experiments, hemodynamic data were digitalized and analyzed using the data acquisition software HEM v3.5 (Notocord, Croissy-sur-Seine, France).

### Experimental protocol

After surgical preparation and stabilisation, pigs were randomly divided into 4 groups. In the Control group, animals were submitted to 12 h of mechanical ventilation and subsequent weaning and awakening without any other treatment. In the other groups, animals received a static intratracheal administration of 15 ml/kg of PFD (C_10_F_18_; F_2_Chemicals®, Preston, Lancashire, UK) or PFOC (C_8_F_18_; F_2_-Chemicals®, Preston, Lancashire, UK) or PFOB (C_8_F_17_Br, OriGen®, Helsingborg, Sweden). These PFC were infused using a catheter into the endotracheal tube without interrupting mechanical ventilation. The volume of 15 ml/kg was chosen as the average volume used in clinical trials with partial liquid ventilation [14]. In order to facilitate perfluorocarbon spreading within the lungs, the filling was separated in 3 consecutive administrations of 5 ml/kg while the animal was placed in the right, supine and left positions, respectively. Prior to any administration, PFC were bubbled with 100% O_2_ for 5 min. In all groups, animals were mechanically ventilated during 12 h under anaesthesia before subsequent weaning and awakening. Ventilatory parameters were maintained as previously described (FiO_2_ = 30%; Respiratory rate and tidal volume = 17 cycles/min and ≈ 330-350 ml/cycle, respectively). Animals then returned to their cages for a daily follow-up during a total duration of 96 h after PFC administration. Hemodynamic parameters and blood gases were assessed under anaesthesia throughout the initial procedure, as well as at t = 48 h and 96 h in awake animals. After the last recording (at t = 96 h), animals were euthanized by an overdose of pentobarbital (150 mg/kg i.v.) for organ sampling.

### Pathology and histology

After euthanasia, a complete autopsy of each animal was performed. All analyses were systematically blinded for group allocation. Lungs were removed, photographed and each lobe was sampled for subsequent analyses. Samples were fixed by formaldehyde and stored for the assessment of residual PFC contents, as described in the next section (one section for each lobe = 6 sections for each pig). They were subsequently prepared for histological analyses using haematoxylin–eosin-saffron staining. Beyond the description of any pathological appearance, the pathologist attributed a 0 to 10 semi-quantitative score to the most common lesions observed in these samples*, i.e*., abnormal alveolar wall thickening and alveolar infiltration with foam macrophages (0 = normal, 10 = very severe lesion). A score was attributed for each lobe and a mean lung score was then calculated for each animal.

### Magnetic resonance imaging (MRI)

In order to assess the amount of residual PFC within the lung, formaldehyde-fixed samples were studied using fluorine MRI with a 1.5 T system equipped with the multi-nuclei option (Achieva, Philips, Best, The Netherlands). Dedicated transmit-receive radiofrequency switch and pre-amplifier tuned to ^19^ F frequency were designed. A two-port dual-resonant volume coil comprising a 10-cm diameter Helmholtz coil (^19^ F) and a 10-cm long saddle coil (^1^H) were built and connected to transmit-receive switches. For each pig, the seven lobe samples were imaged at the same time and 3 samples of pure PFD, PFOC and PFOB were added in the field-of-view of the coil for signal calibration and quantification. For parenchyma imaging, a 3D spoiled gradient echo sequence with 1 mm isotropic resolution, TR/TE = 8.1/3.7 ms, bandwidth-per-pixel 191 Hz, matrix size 164×72×72, was applied to obtain T1-weigthed (flip angle 20°, 1 average) and proton density (flip angle 5°, 8 averages) scans. To evaluate PFC, fluorine images were acquired using a 3D spoiled gradient echo sequence with isotropic 1.5 mm resolution, TR/TE = 12/2.9 ms, bandwidth-per-pixel 2.2 kHz, matrix size 116×52×52, flip angle 10° and 16 averages. Bandwidth and echo time were chosen such that chemical shift artifact was minimized and that the various peaks of the complex spectra were approximately in phase for all PFC used.

Each sample was segmented in 3D by manually tracing regions-of-interest (ROI) using the T1W and proton density images. The ROI were used to evaluate the lung sample volumes and were reported onto the fluorine images for PFC quantification. ROI were traced over the tubes containing pure PFCs in the ^19^ F images and the mean signal was calculated. Noise was measured as the standard deviation in a region void of signal. Fluorine images were thresholded at 3 times the noise level and converted to concentration using the adequate reference PFC signal from the tubes. The mean concentration within each sample was then estimated.

### Statistical analysis

Parametric data were expressed as mean ± SEM. Non parametric data were expressed as median and individual values. Hemodynamic parameters, temperature and blood pH and gases partial pressure were considered as parametric data. They were compared between groups throughout the protocol using a two-way analysis of variance for repeated measures. In order to avoid multiple comparisons, post-hoc analyses were only made between groups but not among different time-points. Accordingly, when the global analysis did not show a significant group effect or an interaction between time and groups, we did not perform post-hoc analysis. When a group effect was significant, we always had a significant interaction between time and groups. Therefore, we performed post-hoc analysis at each time-point between groups, but not between the different time-points in a single group. Histological scores and lung concentrations of PFC assessed by MRI were considered as non parametric data. They were compared between groups using a non-parametric Kruskall-Wallis analysis. The level of significance was set at p < 0.05.

## Results

### Number of animals, hemodynamic and respiratory parameters

Sixteen animals were included in the present study, *i.e.*, 4 pigs in each group. They all completed the protocol, except one animal which experienced severe respiratory failure after awakening in the PFD group. This pig was prematurely euthanized at 24 h for ethical reasons and its lung samples were analyzed using the same procedures than those used for other animals (fluorine MRI and histology). As shown in Table 1, heart rate, mean arterial pressure, rectal and esophageal temperatures were not different among groups throughout the follow-up.

**Table 1:** Hemodynamic parameters and temperatures throughout protocol

	**Baseline**	**Time after perfluorocarbon administration (h)**														
		**1**	**12**	**48**	**96**											
*Number of animals*																
Control	4	4	4	4	4											
PFD	4	4	4	3*	3*											
PFOC	4	4	4	4	4											
PFOB	4	4	4	4	4											
*Body weight (kg)*																
Control	23.9 ± 0.5	-	-	-	23.9 ± 1.1											
PFD	25.1 ± 2.0	-	-	-	22.8 ± 1.0											
PFOC	24.5 ± 0.8	-	-	-	24.0 ± 0.8											
PFOB	22.2 ± 1.2	-	-	-	22.4 ± 0.6											
*Heart rate (beats/min)*																
Control	115 ± 7	106 ± 4	87 ± 3	103 ± 3	112 ± 9											
PFD	121 ± 7	114 ± 7	99 ± 5	116 ± 9	115 ± 7											
PFOC	121 ± 5	120 ± 5	96 ± 4	110 ± 9	114 ± 7											
PFOB	121 ± 18	119 ± 17	96 ± 9	115 ± 12	113 ± 10											
*Mean arterial pressure (mmHg)*																
Control	82 ± 5	81 ± 5	79 ± 7	91 ± 3	77 ± 5											
PFD	87 ± 7	85 ± 5	82 ± 5	91 ± 10	101 ± 12											
PFOC	81 ± 6	77 ± 3	72 ± 2	97 ± 12	97 ± 3											
PFOB	77 ± 4	75 ± 2	75 ± 5	99 ± 8	94 ± 7											
*Rectal temperature (°C)*																
Control	38.2 ± 0.2	38.2 ± 0.1	38.2 ± 0.1	38.6 ± 0.4	38.2 ± 0.3											
PFD	37.8 ± 0.1	37.7 ± 0.2	38.2 ± 0.2	38.3 ± 0.3	37.9 ± 0.2											
PFOC	37.8 ± 0.1	38.0 ± 0.2	38.2 ± 0.1	38.6 ± 0.1	38.5 ± 0.3											
PFOB	38.1 ± 0.1	38.1 ± 0.1	38.2 ± 0.1	38.6 ± 0.1	38.5 ± 0.2											
*Esophageal temperature (°C)*																
Control	37.8 ± 0.1	38.18 ± 0.1	38.1 ± 0.1	-	-											
PFD	37.7 ± 0.2	37.65 ± 0.3	38.1 ± 0.3	-	-											
PFOC	37.7 ± 0.1	37.8 ± 0.2	38.1 ± 0.2	-	-											
PFOB	38.1 ± 0.1	38.08 ± 0.1	38.4 ± 0.1	-	-											

PFD, perfluorodecalin; PFOC, perfluoro-N-octane; PFOB, perfluorooctylbromide; *, One animal was euthanized for respiratory distress at t = 24 h in the PFD group.

At baseline, the animals had similar blood pH and partial pressure for O_2_ and CO_2_ (Table 2). Ventilator parameters were similar among groups (minute volume = 0.30 ± 0.03, 0.26 ± 0.03, 0.26 ± 0.01 and 0.31 ± 0.01 ml/min/kg in the Control, PFD, PFOC and PFOB groups, respectively). After PFC administration (t = 1 h), a transient decrease in PaO_2_ and PaC0_2_ were observed in the PFOB and PFOC groups as compared to Control. At t = 12 h, PaO_2_ values were significantly decreased in the PFD group as compared to Control and PFOC groups. One animal of the PFD group was even more hypoxemic after awakening (at t = 24 h, PaO_2_ and PaCO_2_ = 22 and 60 mmHg, respectively). As mentioned above, this animal was euthanized for ethical reasons before completing the protocol. The surviving animals in the PFD group also elicited abnormal blood gases with a significant increase in PaCO_2_ at t = 96 h. In the PFOB and PFOC groups, blood gases after awakening and subsequent follow-up were not significantly altered as compared to the Control group (t = 48 and 96 h).

**Table 2:** Blood pH and gases in the different groups

	**Baseline (Anesthetized)**	**Time after perfluorocarbon administration (h)**														
		**1 (Anesthetized)**	**12 (Anesthetized)**	**48 (Awake)**	**96 (Awake)**											
*Number of animals (n)*																
Control	4	4	4	4	4											
PFD	4	4	4	3*	3*											
PFOC	4	4	4	4	4											
PFOB	4	4	4	4	4											
*Blood pH*																
Control	7.45 ± 0.04	7.43 ± 0.03	7.42 ± 0.02	7.37 ± 0.05	7.36 ± 0.02											
PFD	7.47 ± 0.02	7.45 ± 0.04	7.43 ± 0.03	7.29 ± 0.11	7.23 ± 0.09											
PFOC	7.47 ± 0.01	7.57 ± 0.02	7.45 ± 0.03	7.31 ± 0.07	7.36 ± 0.03											
PFOB	7.45 ± 0.01	7.53 ± 0.02	7.40 ± 0.03	7.32 ± 0.05	7.38 ± 0.04											
*pO*_*2*_*(mmHg)*																
Control	126 ± 6	118 ± 8	115 ± 4	103 ± 2	103 ± 2											
PFD	146 ± 4	98 ± 7	90 ± 13^†^	84 ± 11	79 ± 9											
PFOC	137 ± 4	100 ± 4	113 ± 8^‡^	95 ± 3	93 ± 5											
PFOB	125 ± 3	82 ± 5^†^	95 ± 12	92 ± 9	95 ± 6											
*pCO*_*2*_*(mmHg)*																
Control	43 ± 4	45 ± 2	47 ± 4	40 ± 3	38 ± 2											
PFD	40 ± 3	43 ± 7	43 ± 4	44 ± 2	57 ± 11^†^											
PFOC	37 ± 2	30 ± 2^†^^‡^	43 ± 4	43 ± 2	43 ± 2											
PFOB	40 ± 1	35 ± 1	46 ± 5	40 ± 2	43 ± 5											

PFD, perflurodecalin; PFOC, perfluoro-N-octane; PFOB, perfluorooctylbromide; *, One animal was euthanized for respiratory distress at t = 24 h in the PFD group; ^†^p < 0.05 vs corresponding value in the Control group; ^‡^p < 0.05 vs corresponding value in the PFD group.

### Assessment of lung residual PFC with MRI

**Figure 1:**
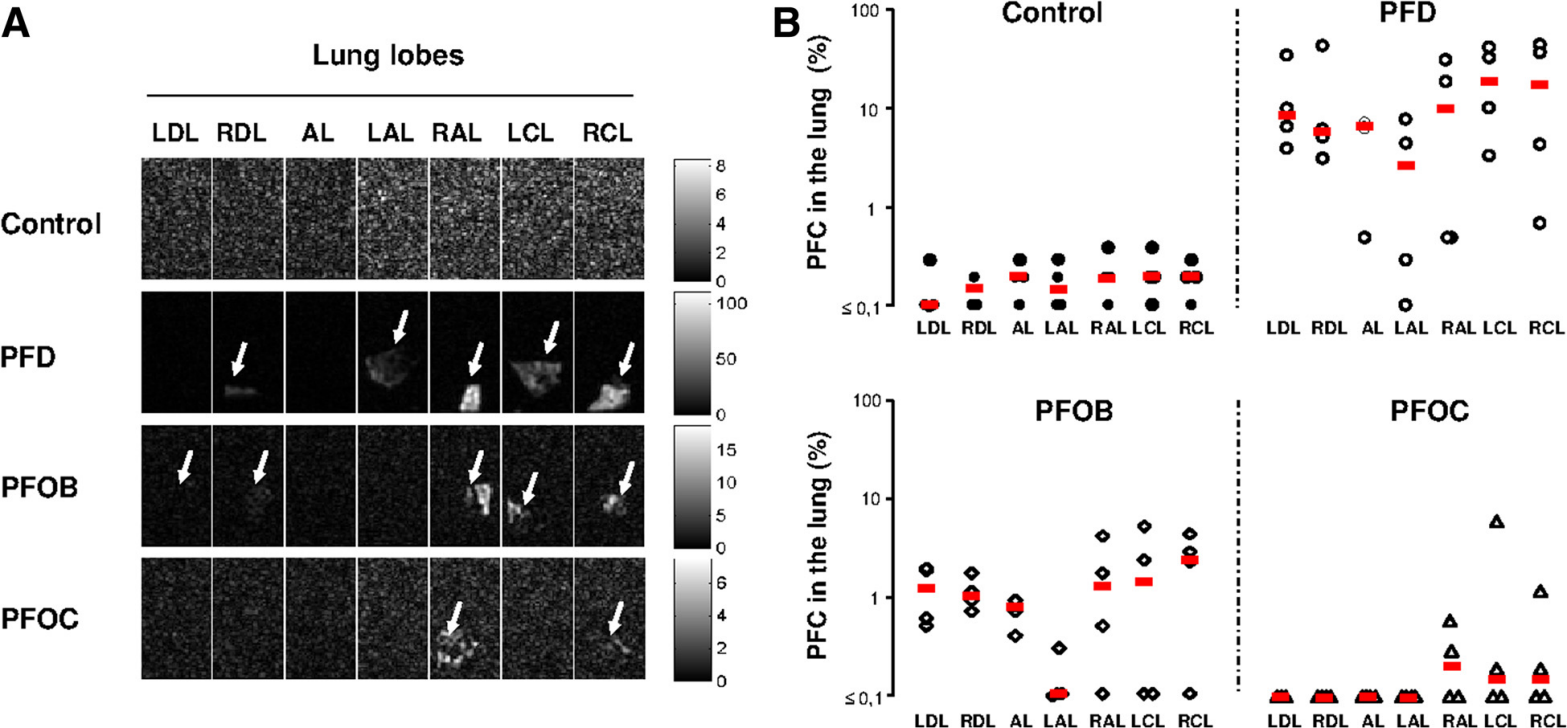
**Assessment of pulmonary residual perfluorocarbons using **^**19**^ **F Magnetic Resonance Imaging (MRI). ***Panel****A***: Typical imaging of lung samples from the different lobes with ^19^ F MRI. For each group, a set of pictures shows images from the same animal. The bright areas represent the voxels filled with perfluorocarbon. Arrows show lung lobes with residual perfluorocarbons. *Panel****B***: MRI-assessed concentration of perfluorocarbons in each lung samples as percentage of sample volume. Open symbols represent individual values for each sample. Thick lines represent median values in each group. Abbreviations: PFD, perfluorodecalin; PFOB, perfluorooctylbromide; PFOC, perfluoro-N-octane; *, p < 0.05 vs corresponding value in the Control group; LDL, left diaphragmatic lobe; RDL, right diaphragmatic lobe; AL, accessory lobe; LAL, left apical lobe; RAL, right apical lobe; LCL, left cardiac lobe; RCL, right cardiac lobe.

Figure 1A illustrates typical images of fluorine MRI in an animal of each group. An image for each lung lobe is provided, which corresponds to a central slice within each sample. As expected, no signal was detected in the control animal and only noise can be seen. The samples from the PFD group are easily identified (arrows) with a large quantity of signal in almost every lobe. The samples from the PFOB group has detectable amount of signal in several lobes. The PFO group samples only displayed limited PFC traces. MRI data post-processing provided the mean concentration of each PFC within each lung sample. As illustrated in Figure 1B, the amount of residual PFC was very high in all samples from the PFD group, averaging ~10% of lung volume. This percentage was lower in the PFOB group (~1%) but remained significant as compared to Control values. In the PFOC group, the percentage of residual PFC was not significant when compared to the Control group (~0.1%, *i.e.,* on the order of the noise level observed in Control samples).

### Lung histology

**Figure 2:**
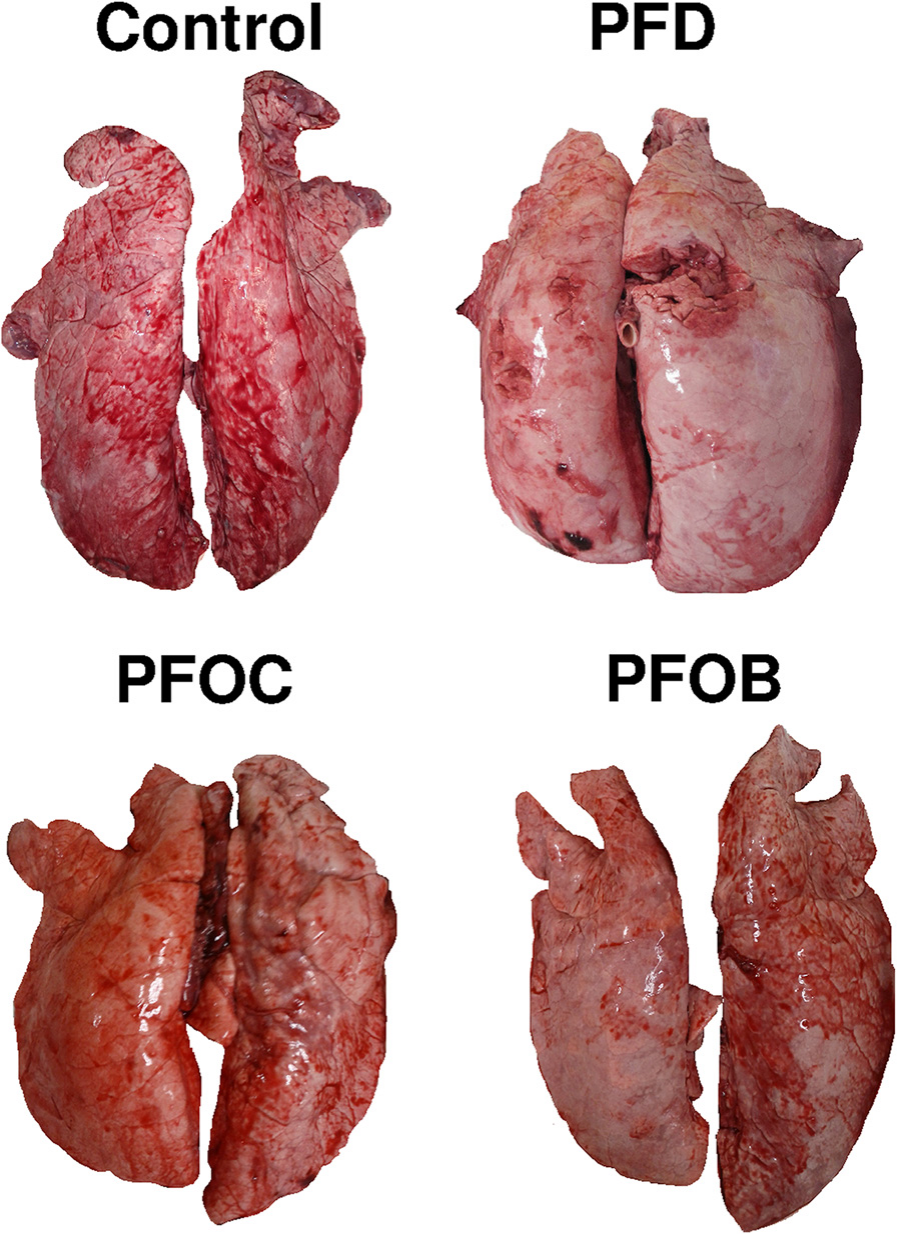
**Macroscopic appearance of the lungs in the different groups.** Appearance was normal in the Control group, while lungs appeared distended in the PFD group. Intermediate features can be seen for PFOB and PFOC groups. Abbreviations: PFD, perfluorodecalin; PFOB, perfluorooctylbromide; PFOC, perfluoro-N-octane.

**Figure 3:**
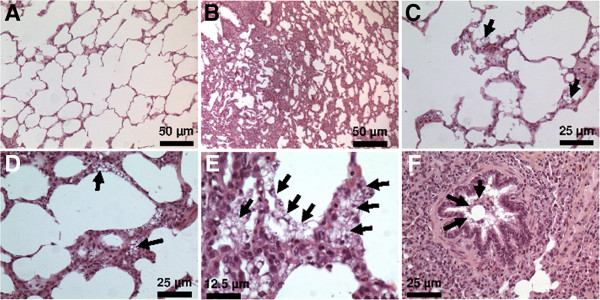
**Histological appearance of the lungs in the different groups.** Panel **A**: Normal appearance of the lung in a sample from the PFOC group. Panel **B**: Mild alveolar wall thickening in a sample from the PFOB group. Panel **C**: Mild infiltration by foam macrophages (arrows) in a sample from the PFD group. Panel **D**: Mild alveolar wall thickening in a sample from the PFOB group, with concomitant infiltration by foam macrophages (arrows). Panel **E**: Alveolar infiltration by foam macrophages (arrows) in a sample from the PFOB group. Panel **F**: Foam macrophages (arrows) within a bronchiole in a sample from the PFOB group. Abbreviations: PFD, perfluorodecalin; PFOB, perfluorooctylbromide; PFOC, perfluoro-N-octane.

**Figure 4:**
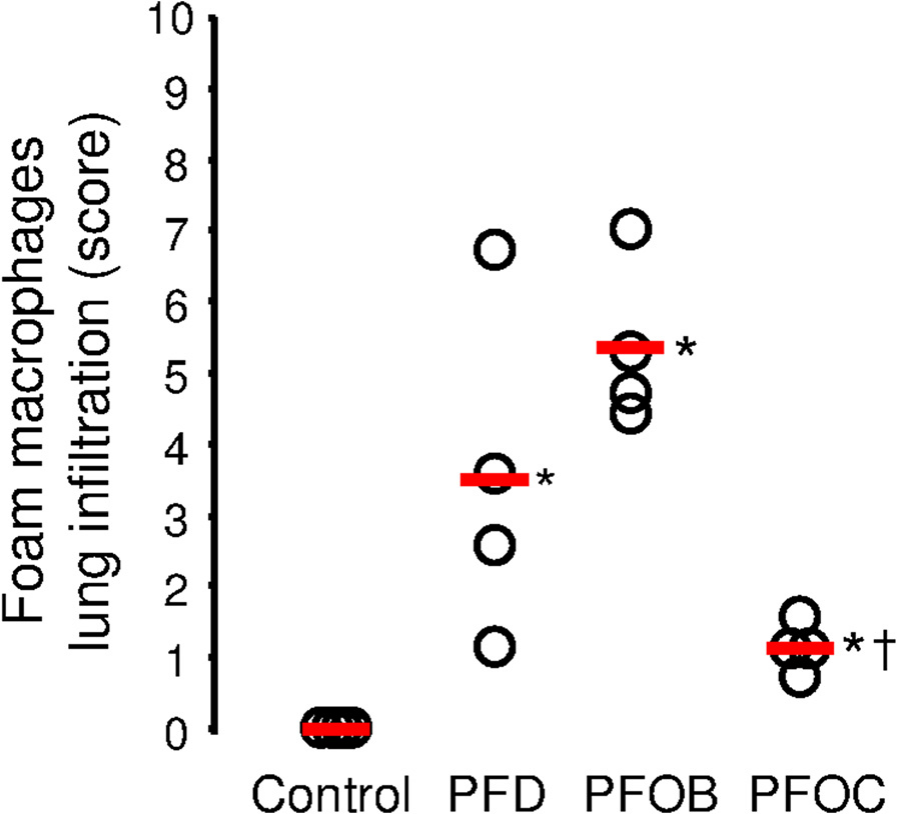
**Histological score of foam macrophages lung infiltration in the different groups.** Open symbols represent individual values for each sample. Thick lines represent median values in each group. Abbreviations: PFD, perfluorodecalin; PFOB, perfluorooctylbromide; PFOC, perfluoro-N-octane; *, p < 0.05 vs corresponding value in the Control group; †, p < 0.05 vs corresponding value in the PFOB group.

Macroscopic examinations of the lungs revealed an apparent overdistension in the groups treated with PFC as compared to the Control group (Figure 2). This observation was not quantified but appeared to be dramatic in the PFD group and only very mild in the PFOB and PFOC groups. The blind analyses of histological slices further revealed alveolar wall thickening in several samples, probably as a consequence of the mechanical ventilation episode. This alteration was not related to PFC treatment as it was also often observed in the Control group. As an example, Figures 3A and B illustrate the normal appearance of the lung in a sample from the PFOC group as compared to a mild alveolar wall thickening showing some hypercellularity but no fibrosis in the PFOB group. However, these lesions were mild in most samples. The most striking feature was rather an alveolar infiltration with foam macrophages as illustrated in samples from the PFD and PFOB groups in Figures 3C, D and E (arrows). In a few samples, foam macrophages were even identified in bronchioles (*e.g*., Figure 3F from the PFOB group). In order to compare the severity of macrophage infiltration among groups, a semi-quantitative score was blindly attributed to each animal using the mean value of the different lobes. As illustrated in Figure 4, foam macrophage infiltration was only observed in the groups treated by PFC but not in the Control group. It was more pronounced in the PFD and PFOB groups as compared to the PFOC group.

## Discussion

This study demonstrates that static administration of PFOB or PFOC did not compromise gas exchanges after rapid weaning from mechanical ventilation whereas PFD was poorly tolerated and led to severe hypoxemia in pigs. In order to link lung recovery and PFC residues, we assessed simultaneously lung histology and PFC concentrations using fluorine MRI on lung samples. This imaging tool is highly specific and can be used with all PFC. The poor tolerance of PFD in the present study was probably related to the large amount of residual PFC while PFOB was present at only low levels within the lungs. In comparison, PFOC was virtually eliminated from the lungs after 12 h of mechanical ventilation and 4 days of recovery. This is in agreement with a previous report in humans experiencing liquid ventilation with PFOB and showing residual detectable PFC levels at radiography during at least 4 days after the last administration [26].

The rapid elimination of PFOC can be explained by its high vapour pressure and evaporative properties as compared to PFOB and PFD (vapour pressure ≈ 50 vs 10.4 and 13.6 mmHg at 37°C, respectively) [27,28]. Conversely, differences in vapour pressures do not explain the amount of residual PFC in the PFD group as compared to PFOB. As described by Faithfull et al. [29], PFC distribution actually depends on the interface between PFC and both gas and aqueous phases during liquid ventilation. Depending on the surface tensions between PFC and both air and water, a PFC could either become “wettable” and widely distributed or conversely form droplet and be poorly distributed. Faithfull et al. [29] calculated the spreading coefficient of several PFC and demonstrated that positive values (good “wettibility”) can be obtained with PFOB (+2.7 dyn/cm at 25°C) whereas negative values were obtained with PFD (−1.5 dyn/cm at 25°C). Using the surface tensions calculated by Meinert and Roy for PFOC, we also estimated the spreading coefficient of PFOC to +3.0 dyn/cm at 25°C [30]. It means that PFOB and PFOC can be distributed homogenously at the interface between air and water while PFD tends to form droplets. The more homogeneous distribution of PFOB was confirmed by tomography in rabbits as compared to PFD [13]. These phenomenona can also explain air trapping and formation of menisci of PFD within lung airways. This could be linked to the severe hypoxia and apparent lung overdistension observed in the present study in the PFD group. Such overdistension can also be induced or worsened by the mechanical ventilation after PFC administration. Indeed, one can argue that our protocol used constant tidal volumes during mechanical ventilation even after PFC administration, as previously done in most trials with partial liquid ventilation. Ricard et al. showed that such a strategy combined with high doses of PFOB can worsen ventilation-induced lung injury of healthy [31] or preinjured lungs [32], respectively. This may provide some explanation for the negative results of the large-scale clinical trial conducted with partial liquid ventilation in adults with acute respiratory distress syndrome [14]. It might be more relevant to temporally decrease tidal volumes after PFC administration in order to prevent putative trauma inducing secondary distension, until sufficient evaporation of the PFC.

The results regarding PFC residual concentrations were corroborated by histological analyses. Indeed we observed numerous foam macrophages within alveoli and/or airways in PFOB and PFD groups, probably as a result of PFC clearance. In accordance with the low concentrations of PFC residues in the PFOC group, we observed only few foam macrophages in this group. Indeed, such histological appearance was likely expected as macrophages are well known to form vacuoles and become foamy in the presence of PFC [33,34]. Lung foam macrophages were observed in previous reports, *e.g.,* in rabbits submitted to total liquid ventilation with PFD [22] or after intravenous administration of PFC through secondary lung elimination [34]. It was also proposed that pigs could be highly sensitive to PFC-induced macrophage activation through a high concentration of alveolar resident macrophages in comparison to other species. As an example, total liquid ventilation with PFD was much better tolerated in rabbits [10,13,22] and lambs [3]. The present study suggests, however, that even in pigs, specific PFCs may be well tolerated, while others will be toxic. Pigs could therefore be an appropriate species for safety experiments with PFC but for studies of treatment effects (e.g., resuscitation studies), it will depend on the specific PFC used. From these observations, it is also clear that PFOC could be well tolerated and rapidly eliminated when a very rapid weaning/awakening is expected after PFC administration, while PFOB might be more relevant for longer term mechanical ventilation. As a limitation of our study, we should however emphasize that we only followed animals during a short period (4 days). Previous trials investigated this aspect in other conditions, *e.g.*, with perfluorocarbon nanoemulsion [35].

Finally, our study also showed the importance of fluorine MRI for PFC imaging. This technique offers promising perspectives for general lung imaging using fluorinated gases or liquids [25], and especially for clinical testing with liquid ventilation. As previously demonstrated [36,37], this is a highly specific technique that could be a useful additional tool before taking any decision of patient weaning of ventilation support.

## Conclusions

In conclusion, intrapulmonary administration of PFOB and PFOC appeared to be well tolerated regarding respiratory function and gas exchanges after awakening in pigs. PFOC was rapidly cleared from the lung and virtually disappeared after 4 days whereas PFOB persisted at significant levels and led to foam macrophage infiltration. PFOC could be relevant in the setting of intrapulmonary administration for short durations, *e.g.*, for total liquid ventilation induced-cooling. In comparison, PFD was poorly tolerated and compromised gas exchange, probably as a consequence of large PFC residues.

## Abbreviations

MRI: Magnetic resonnance imaging; PFC: Perfluorocarbon; PFD: Perfluorodecaline; PFOB: Perfluorooctyl bromide; PFOC: Perfluoro-N-octane.

## Competing interest

R. Tissier and A. Berdeaux are named as inventors in a patent application (Method and System for Treatment of a Body of a Mammal in Cardiac Arrest. U.S. Patent application 20120226337).

The authors declare that they have no competing interests.

## Authors’ contribution

The following authors participated in the *in vivo* investigations: CM; LF; KM; SA; GB; RJD; BA; TR. The following authors performed the pathological analyses: BP. The following authors performed the magnetic resonance imaging method validation and experiments: DL, DRL, MX. The following authors elaborated and participated to the MRI experiments: DRL, BYA, CM. The following authors participated in protocol conception and/or data analyses: BP; CM, DL, DRL, GB; ID, MX, RJD; BA; TR. All authors participated in the manuscript preparation and correction. All authors read and approved the final manuscript.

## Pre-publication history

The pre-publication history for this paper can be accessed here:

http://www.biomedcentral.com/2050-6511/15/53/prepub

## Supplementary Material

Additional file 1 The ARRIVE guidelines checklist. Click here for file
